# ANALYSIS OF CIRCADIAN TRANSCRIPTION ACTIVATOR BMAL1 AND CLOCK BINDING TARGETS IN YOUNG AND OLD MOUSE SKELETAL MUSCLE

**DOI:** 10.1093/geroni/igad104.2480

**Published:** 2023-12-21

**Authors:** Xiping Zhang, Mark Viggars, Karyn Esser

**Affiliations:** University of Florida, Gainesville, Florida, United States; University of Florida, Gainesville, Florida, United States; University of Florida, Gainesville, Florida, United States

## Abstract

Circadian rhythms play important roles in many physiological processes and circadian rhythm disturbances are often observed in older people. Circadian rhythms are regulated by a molecular clock mechanism that is found in virtually all cells of the body. The core clock mechanism consists of transcriptional activators (BMAL1 and CLOCK) and the feedback negative regulators (PER and CRY). Beyond keeping daily time, the core clock factors regulate a daily gene expression program and this daily program is diminished with age. Skeletal muscle is critical in supporting systemic metabolism and mobility. Loss of muscle strength in older people are related to increased morbidity and mortality. Our previous studies have shown a significant decline of circadian genes with age in skeletal muscle and there are very few circadian genes in common between young and old mouse muscle. To understand the mechanism of the diminished circadian gene expression with age, we performed ChIPseq to determine BMAL1 and CLOCK binding and ATACseq to determine chromatin accessibility with muscle from young (6 months) and old (27 months) mouse. The ChIPseq result showed that BMAL1 and CLOCK shared more than 8,000 binding peaks in young muscle and less than 200 binding peaks in old muscle. The ATACseq identified that chromatin accessibility altered in more than 7000 sites with age and BMAL1:CLOCK binding sites were less accessible with age. In conclusion, our research has demonstrated that muscle chromatin accessibility changes with age and the core clock factors are important regulators of gene expression in skeletal muscle.

